# Extended Neuroendoscopic Endonasal Approach for Resection of Craniopharyngioma in Children

**DOI:** 10.3389/fneur.2022.771236

**Published:** 2022-01-31

**Authors:** Danyang Wu, Ling Xu, Sungel Xie, Feiji Sun, Mingxiang Xie, Pei Wang, Shunwu Xiao

**Affiliations:** ^1^Department of Neurosurgery, The Affiliated Hospital of Zunyi Medical University, Zunyi, China; ^2^Graduate School, Zunyi Medical University, Zunyi, China

**Keywords:** craniopharyngioma, children, surgical technique, neuroendoscopy, endonasal approach

## Abstract

**Objective:**

To explore the surgical approach and technique of neuroendoscopic endonasal resection of pediatric craniopharyngiomas and to further evaluate its safety and effect in children.

**Methods:**

The clinical data of 8 children with craniopharyngiomas who were surgically treated by neuroendoscopy through an extended endonasal approach in our center from 2018 to 2021 were retrospectively analyzed. The related surgical approach and technique were evaluated to improve the surgical results and further reduce the surgical complications when removing craniopharyngioma in children.

**Results:**

All 8 patients achieved a gross-total resection of the tumor under neuroendoscopy. Postoperatively, 2 cases of transient hyperthermia and 4 cases of transient hyper- and/or hyponatremia occurred within the first 2 weeks, all of which were quickly controlled. Seven patients had symptoms of diabetes insipidus to varying degrees after the operation, and 4 of them improved within 1–3 months after surgery, but 3 cases still needed oral pituitrin. There were no cases of coma or death, leakage of cerebrospinal fluid, or severe electrolyte imbalance after surgery. During the postoperative follow-up of 3 months to 2 years, no tumor recurrence was found. Among the 7 patients who suffered postoperative neuroendocrine deficiencies, 3 patients were found to be temporary during the follow-up, but 4 patients still required hormone replacement therapy. Particularly, postoperative visual deterioration and olfactory defect that occurred in patients were all improved during follow-up periods. In addition, 4 cases of obesity were noted at the last follow-up.

**Conclusions:**

Extended neuroendoscopic endonasal resection of craniopharyngiomas may be used as a safe and effective approach for children. Due to the poor pneumatization of the sphenoid sinus and worse compliance of treatment in children, surgical techniques of exposing the sellar region, removing the tumor, and reconstructing the skull base, as well as postoperative management of patients was proposed. However, due to the limited surgical cases in the study, the surgical safety and effects of the extended neuroendoscopic endonasal approach for children with craniopharyngiomas need to be further studied in the future.

## Introduction

Craniopharyngiomas are a rare and mostly benign epithelial tumor occurring in the sellar and suprasellar regions, which usually involve children and adolescents (1–3). Craniopharyngiomas are deeply located and tend to adhere to circumjacent neurovascular structures, such as optic nerves, hypothalamus, and pituitary stalk (1, 2). Thus, how to safely remove the tumor is a huge challenge for most neurosurgeons. Traditional microscopic surgery for craniopharyngiomas has been well-established and includes the pterion approach, subfrontal approach, presigmoid approach, and interhemispheric transcorpus callosal ventricular approach (4–8). However, these surgical approaches are almost from various supratentorial routes and each route has different degrees or angles of surgical blind areas, especially when operating on intrasellar or infradiaphragmatic tumors (6, 7, 9, 10).

Recently, with the gradual development and application of neuroendoscopy in adult craniopharyngiomas, neuroendoscopy has gradually been used for pediatrics with different tumor characteristics and different ages, which to some extent complement the defects of the simple transcranial microsurgery for craniopharyngiomas (10–12). Although some progress has been made, there are still insufficient reports in the literature for the treatment of children *via* extended neuroendoscopic endonasal resection of craniopharyngioma (6, 10, 13). Therefore, in this study, we would retrospectively summarize the surgical cases of pediatric craniopharyngioma in our center to further explore the surgical approach and technique of neuroendoscopic endonasal resection of craniopharyngioma in children and to evaluate its surgical safety and effect as well as the postoperative management of children with craniopharyngioma.

## Materials and Methods

### Selection of Patients

The data of patients with craniopharyngioma who were surgically treated by extended neuroendoscopic endonasal approach at the Affiliated Hospital of Zunyi Medical University from 2018 to 2021 were retrospectively reviewed. Patients with age under 18 years were included in this study. A total of 8 children were diagnosed with craniopharyngiomas by imaging and pathology, with a median age of 9.5 years (range, 6–18 years). The main clinical manifestations include headache in 8 cases, vomiting in 3 cases, blurred vision or visual field defect in 6 cases, polydipsia and polyuria in 4 cases, and growth retardation in 5 cases.

### Preoperative Examinations and Evaluations

Before the operation, all children underwent head CT examination, especially the three-dimensional CT under the thin-layer scan of the sphenoidal sella and the head CT angiogram (CTA) examination, and the head MRI with enhanced scan. The CT scans were used to understand the tumor calcification, the development or pneumatization of the sphenoid sinus, and whether there is an unruptured aneurysm in the intracranial arterial ring or whether the artery is pushed or wrapped by the tumor from CTA. The head MRI examination was to understand the tumor size, cyst changes, anatomical relationship with surrounding structures, or with or without hydrocephalus, etc. As regards the tumor location, one case was an intrasellar type, and 3 cases were intrasellar-suprasellar type, and 4 cases were suprasellar–ventricular type; among them, 4 cases were with poor pneumatization of sphenoid sinus, and 2 cases were with a turbinal sella (completely no pneumatization). All tumors were solid-cystic and calcified to varying degrees, and 5 cases suffered obstructive hydrocephalus. In this study, we calculated the longest diameter of the tumor to represent the tumor size, with the median diameter of 4 cm (range, 3–5 cm).

Preoperative laboratory examination revealed that 3 patients had reduced gonadal hormones, and 4 patients had low levels of growth hormone. Preoperative visual acuity and visual field examination revealed that 5 patients had decreased vision, and 7 patients had visual field defects. However, the neuropsychological and intelligent evaluations were only performed in one child, therefore, we did not show the data in the study.

The radiological criteria used for the selection of the trans-nasal approach: (1) The main body of the tumor grows along the midline; (2) The lateral growth of the tumor cannot exceed the radiating area by 30° endoscope (with a 30° angle of the intracranial tube opening of the optic canal on the coronal MRI); (3) In addition, a turbinal sella (completely no pneumatization), combined hydrocephalus, and tumor calcification are not contraindications for this trans-nasal approach.

### Surgical Techniques and Procedures

**Figure 1:**
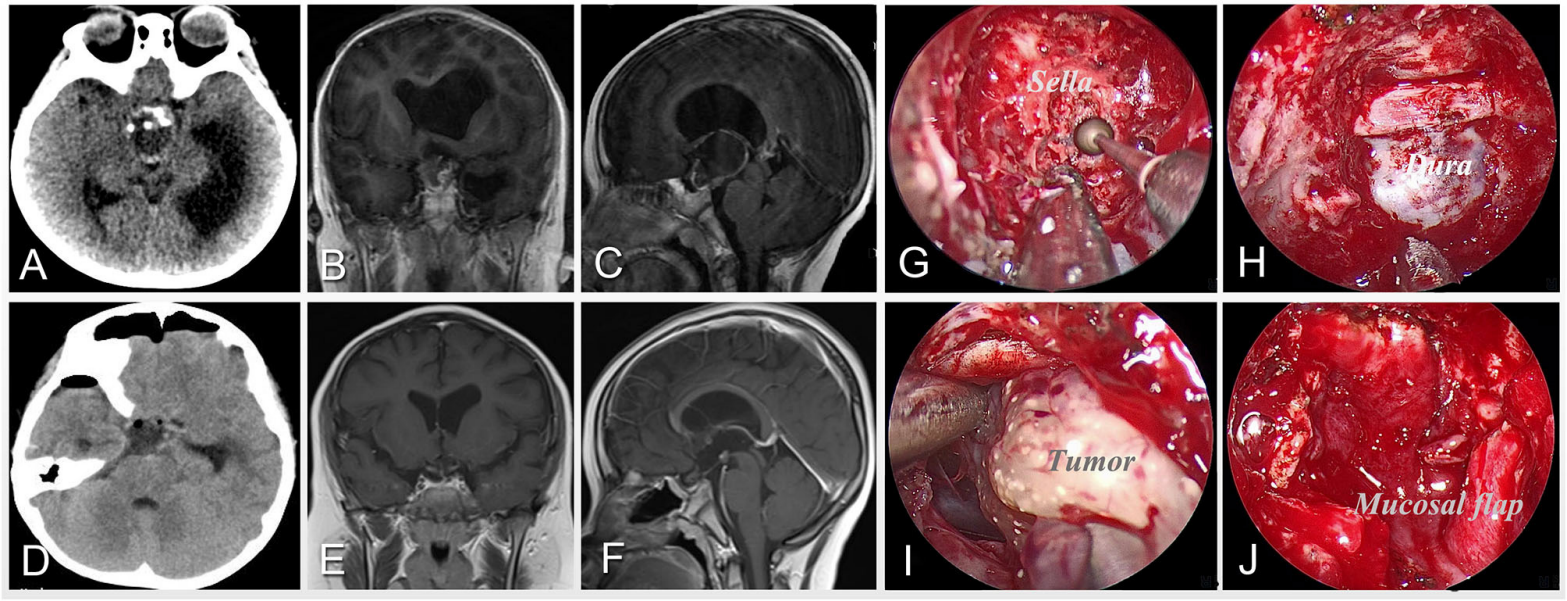
Case 5 was an 8-year-old boy who presented with developmental retardation and headache before surgery. Preoperative imaging of CT **(A)** and MRI **(B,C)** shows a calcified and solid-cystic lesion located in the suprasellar region and invading the third ventricle with obstructive hydrocephalus. After careful preoperative evaluation, the extended neuroendoscopic endonasal approach was performed for the patient. The intraoperative pictures **(G–J)** illustrate the surgical procedures including drilling the sphenoid sella along the midline **(G)**, exposing the bone window for operation **(H)**, and separating the tumor along its boundary **(I)**, and reconstructing the skull base with pedicled mucosal flap after resection **(J)**. Postoperative imaging of CT **(D)** and MRI **(E,F)** demonstrates the gross-total resection of the tumor.

After general anesthesia and endotracheal intubation, the patient was posed as supine, with the head slightly tilted backward and rotated laterally to face the right-side standing surgeon. The three-nail head holder (Mayfield, United States) could be used for children over 8 years of age to fix their heads. A 4-mm, 0° rigid neuroendoscope (Karlstorz, Germany) was used for lighting. Based on both sides of the nasal cavity, we used 1:100,000 adrenaline cotton pads to contract the blood vessels of the bilateral nasal mucosa. Routinely, we removed the right middle and upper turbinate and took the right nasal septum mucosal flap, except that one case was with nasal septum deviating to the right, so the left nasal septum mucosal flap was taken. An electric knife was used to incise the nasal septal mucosa near the right sphenoid crypt. Usually, the incision should avoid the nasal mucosa of the olfactory zone by extending the upper mucosal incision to the nasal vestibule, extending the lower mucosal incision from the posterior nostril and the bottom of the nose to the nasal vestibule, and merging with the upper incision. After peeling the pedicled nasal septum mucosa, it was pulled into the right lower nasal passage for subsequent use. The bony nasal septum was severed, and the left nasal septum mucosa was cut approximately 1.5 cm, and the anterior wall of the bilateral sphenoid sinuses was ground by a drill (Medtronic, United States) to expose the sphenoid sinus cavity. The anatomical landmarks of the sphenoid sinus in children are not always distinguishable, and thus the sellar base should be drilled strictly along the midline to both sides (Figure 1). After exposing the sellar dura mater, expanding the exposure to both sides as well as the upper and lower sides of the sellar floor. Those who are unfamiliar with anatomy in the early stage could use neuronavigation to help to remove the bony compartments of sphenoid sinus, sellar tuberosity, sphenoid plateau, etc. Doppler ultrasound (Hadeco smandop 45, Japan) probe was used to locate the bilateral internal carotid arteries. After that, we electrically coagulated and incised the anterior cavernous sinus, and the bleeding could be stopped with fluid gelatin (Surgiflo, United States). The dura mater of the anterior skull base was incised by 1.5 cm to form a dural window bound by the internal carotid artery above the clinoid process, with the posterior edge exposing one-third or all of the pituitary gland tissue and the anterior edge of exposing the tumor and the optic chiasm.

**Figure 2:**
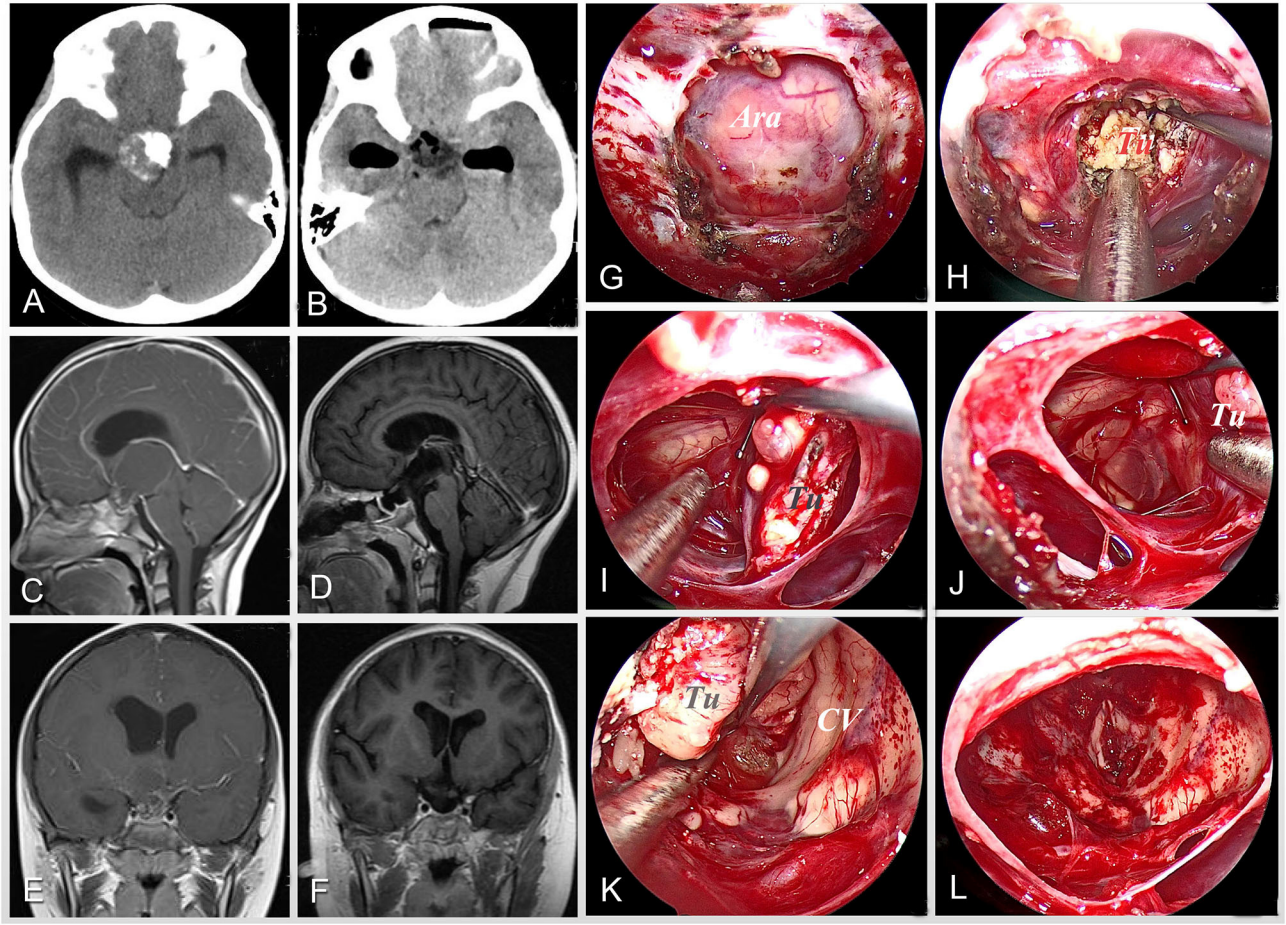
Case 6 was an 8-year-old girl who complained of polyuria, impaired vision, headache, and vomiting on admission. Preoperative images of CT scan **(A)** and MRI scan **(C,E)** show a solid-cystic lesion with obvious calcification, involving the third ventricle with obstructive hydrocephalus. After careful preoperative evaluation, the extended neuroendoscopic endonasal approach was planned for the patient. The intraoperative pictures **(G–L)** illustrate the surgical procedures including exposing the bone window **(G)**, decompressing the tumor by sucking in the intratumor fluid oil **(H)**, separating the tumor along its boundary **(I,J)** and the third ventricular wall **(K)**, and gross-totally removing the tumor **(L)**. Postoperative imaging of CT **(B)** and MRI **(D,F)** demonstrates the complete resection of tumor and relief of hydrocephalus. Tu, tumor; Ara, arachnoid mater; CV, cerebral ventricular.

After fully revealing the operating field for the tumor and the adjacent structures based on preoperative imaging and neuronavigation, the subarachnoid space was opened above the pituitary gland and the superior pituitary artery was slightly pushed aside. First, we explored the pituitary stalk to observe the anatomical relationship between the tumor and the optic nerve, pituitary stalk, and third ventricle (Figure 2). Then, the capsule was incised to decompress the tumor first. If the tumor was calcified, we usually removed the calcified tissue in pieces, with attention to separating and protecting the perforating blood vessels that supply the optic nerve and hypothalamus from the superior pituitary artery. The tumor envelope was then lightly peeled from the optic chiasm and pituitary stalk under a directly close view of the neuroendoscope until it was separated to the gliosis zone at the bottom of the third ventricle. If the tumor is located in the third ventricle, it was necessary to enter the third ventricle through the suprachiasmatic and lamina terminalis. When operating, the papillary body and gray nodules, as well as the pituitary stalk should be protected as much as possible. In some cases of tumors that wrapped pituitary stalk, the pituitary stalk could be dissected early to reduce the difficulty of the operation if it could not be preserved safely.

After the removal of the tumor, the skull base was rebuilt and properly sealed with absorbable artificial meninges and porcine fibrin glue (Johnson & Johnson, United States), and then covered by the previously reserved pedicled nasal septum mucosal flap (Figure 1), with iodoform gauze packed and supported for 12–14 days. Patients without cerebrospinal fluid (CSF) leakage from the nasal cavity did not need lumbar cistern drainage after surgery. Endocrine tests were performed on the first, third, and seventh days after resection to inspect the changes of hypothalamic-pituitary hormones. On the tenth day after the operation, the pituitary MRI scan with the enhanced examination was performed to observe the growth of the mucosal flap as well as the tumor resection. Usually, the third-generation cephalosporin antibiotics were administered to patients for 10–12 days after the operation. The hypophysin was injected intramuscularly and then changed to oral dose to control symptoms of diabetes insipidus in patients after surgery; meanwhile, supplement the cortical and thyroidal hormones, and controlling the intake of salt within 3 days after the operation, with 24-h monitoring of intake-and-output fluid volume, were necessary to maintain the postoperative metabolic balance. Then, 3 days later, the physiological requirements sodium-containing fluids and hormones should be appropriately supplemented, with routinely monitoring of serum electrolyte and pituitary hormone levels as well as routinely preventing epilepsy.

### Follow-Up and Statistical Analysis

All patients were followed up closely after surgery. Follow-up data were collected by telephone and outpatient visits. During the follow-up period, patients were routinely performed with imaging and laboratory examinations and the results were reviewed.

Through a single-center retrospective summary of the clinical data of children with craniopharyngioma, the clinical factors affecting surgery, such as patient age, tumor location and size, and sphenoid sinus development, and the surgical results, including tumor resection extent, postoperative pituitary function, and surgical complications, were evaluated to further optimize the surgical methods and techniques of surgical resection of craniopharyngioma in children. In the study, all quantitative data (such as age and tumor size) were displayed with a median and range, and all qualitative data (such as gender, tumor resection, and incidence of complications) were exhibited with rates. All data analysis was calculated using SPSS 25.0 statistical software.

## Results

All 8 patients achieved a complete tumor resection under the direct sight of neuroendoscope, and postoperative imaging examinations further confirmed the complete resection. In particular, all 8 cases with tumors were histopathologically diagnosed as adamantinomatous. Postoperatively, 3 cases suffered transient hyperthermia, and 3 patients had early mild hypernatremia, and 2 patients had late hyponatremia after surgery, including one case experiencing both early hypernatremia and late hyponatremia, all of which were quickly corrected. Seven patients had symptoms of diabetes insipidus to varying degrees after surgery, including 4 patients with polyuria before the operation; 2 of them improved within 1 week after surgery and returned to normal status at 2 weeks; 3 patients returned to normal within 1–3 months, and 3 patients still needed oral pituitrin therapy. The visual acuity was unchanged in 3 cases and improved in 2 cases, and there was no new case of visual impairment. One patient developed a slight fever after the resection, and the CSF test indicated the possibility of intracranial infection, which was well controlled after positive antibiotic treatment. There were no cases of coma or death, no CSF leakage, and no severe electrolyte disturbance after the operation. All patients did not enter the neuro-intensive care unit after surgery and received postoperative treatment in the general ward.

The 8 patients were followed up after surgery. The follow-up time ranged from 3 months to 2 years, with a median time of 12 months. During the follow-up, no tumor recurrence was found on MRI reexamination. The decreased visual acuity and visual field defects were recovered; 2 patients with hyposmia also improved 3 months after the operation. Six cases encountered postoperative pituitary–thyroid axis hypofunction, of which 3 cases improved within 1–3 months after surgery, and 3 patients still needed extra hormone replacement treatment; patients with pituitary–adrenal axis dysfunction was found in 7 cases, and among them, 3 cases improved to varying degrees, but 4 cases still needed extra oral drug therapy; in addition, 2 cases had hypogonadism and 4 cases had decreased growth hormone. At the last time of follow-up, 4 obese patients were noted (Details are shown in Table 1).

**Table 1:** The detailed data of all 8 cases with craniopharyngiomas, including demographic and tumor characteristics, surgical complications and follow-up outcomes.

**Number**	**Age/y**	**Sex**	**Size/** **cm**	**calcification**	**Tumor location**	**Sphenoid sinus** **(pneumatization)**	**Hydroce** **phalus**	**Surgical Complications**					**Postoperative follow-up outcomes**					
								**Hyperthermia**	**Hyper- or** **hyponatremia**	**CSF leak**	**IC** ** infection**	**Diabetes** ** insipidus**	**Reduced cortin**	**Reduced thyroxin**	**Reduced** ** gonadal** ** hormone**	**Reduced** ** somatotropin**	**Obesity**	**Follow-up/m**
1	6	F	3.5	Y	Third ventricular	Well	Y	N	Y	N	N	N^*^	Y	N^*^	Y	Y	Y	24
2	18	M	4	Y	Suprasellar	Well	N	N	N	N	Y	N^*^	N^*^	N	Y	N	N	16
3	9	M	5	Y	Third ventricular	Poor	Y	Y	Y	N	N	Y	Y	Y	N	Y	Y	19
4	16	M	3.5	Y	Intrasellar	Well	N	N	N	N	N	N	N	N	N	N	N	26
5	8	M	4.2	Y	Third ventricular	Poor/turbinal	Y	Y	N	N	N	Y	Y	Y	N	Y	Y	6
6	8	F	4.5	Y	Third ventricular	Poor/turbinal	Y	Y	Y	N	N	Y	Y	Y	N	Y	Y	8
7	18	M	4	Y	Suprasellar	Well	N	N	Y	N	N	N^*^	N^*^	N^*^	N	N	N	5
8	10	M	3	Y	Suprasellar	Poor	N	N	N	N	N	N^*^	N^*^	N^*^	N	N	N	3

*F, female; M, male; Y, yes; N, no; CSF, cerebrospinal fluid; IC, intracranial; y, year; m, month*.

* *Patients with transient symptoms that occurred in the early postoperative period and gradually improved during the follow up*.

## Discussion

Craniopharyngiomas are benign intracranial tumors originating from embryonic malformations, which are categorized as adamantinomatous or papillary (1, 2). They are commonly found at the sellar or parasellar, suprasellar region, from sella turcica to the third ventricle along with the hypothalamic-pituitary axis (1, 7). The clinical manifestations include pituitary or hypothalamic deficiencies, visual impairment, and increased intracranial pressure, all of which can be attributed to the tumor mass impacting the optic nerve or chiasma, the hypothalamic-pituitary axis, and the CSF circulation (1, 14). Surgical resection of the craniopharyngiomas has been seen as the best choice to remove the mass and to improve symptoms. However, craniopharyngiomas occur in a deep location, adjacent to important endocrine structures of the hypothalamus and pituitary, and visual nerve tracts. How to safely and completely remove the tumor is still confusing for most neurosurgeons (1, 7, 14). Many scholars believe that subtotal resection and adjuvant radiotherapy could be an alternative for the treatment of craniopharyngiomas (8, 14, 15). Thus, the phenomenon of “malignant results of benign tumors” is also common in clinics (15–17).

Traditional microscopic surgery for children with craniopharyngiomas has been well developed and includes various supratentorial approaches (4, 5, 7, 10). However, these approaches are still limited due to their surgical blind areas when operating at sellar or parasellar regions, which may finally compromise to incomplete resection, with residual tumor recurrence and postoperative severe complications caused by increased traction of brain tissue (10, 12). Recently, with great advance and increasing application of neuroendoscopy in adult craniopharyngiomas, neuroendoscopy has gradually been applied to the resection of craniopharyngiomas in children (Table 2) (5, 6, 9, 12). However, there are fewer surgical cases in pediatrics being reported, and the surgical techniques related to postoperative outcomes and complications as well as the postoperative management of pediatrics are not yet well defined (1, 7).

**Table 2:** The summary of surgical cases of pediatric patients with craniopharyngiomas removed by endoscopic trans-nasal approach from the literature since 2010^*^.

**References**	**No. of case**	**GTR (%)**	**Surgical complication (n)**	**Tumor recurrence (%)**	**Mean follow-up period (months)**
Madsen et al. (18)	28	85.7	Hypopituitarism (26), new obesity (6), mental disorder (5), CSF leak (2)	40.0	14
Kim et al. (19)	39	92.3	Decreased vision (1), hypopituitarism (18), aseptic or bacterial meningitis (5), delayed ventricular hemorrhage (1)	15.4	47
Giovannetti et al. (20)	12	100.0	Diabetes insipidus (2), CSF leak (2)	0.0	22
Schelini et al. (21)	20	70.0	CSF leak (1), hypopituitarism (11)	15.0	64
Javadpour et al. (22)	15	60.0	Decreased vision (1), hypopituitarism (10), diabetes insipidus (10), postoperative weight gain (1), mental disorder (1)	13.3	77
Patel et al. (23)	16	93.8	New obesity (6), CSF leak (3), meningitis (1), unilater injury of oculomotor nerve (2), hypopituitarism (7), diabetes insipidus (10)	6.3	56
Koumas et al. (24)	12	75.0	Vasospasm/stroke (3), hydrocephalus (1), new obesity (2), meningitis (1), diabetes insipidus (4), hypopituitarism (5)	0.0	39
Stapleton et al. (25)	20	100.0	CSF leak (4), meningitis (4), hydrocephalus (6), intracranial hemorrhage (1)	35.0	/
Alalade et al. (26)	11	45.0	Decreased vision (5), CSF (1), intracranial infection (1), new obesity (6), hypopituitarism (10), diabetes insipidus (7)	9.0	44
d'Avella et al. (27)	12	75.0	CSF leak (1), hypopituitarism (3), diabetes insipidus (4)	33.3	78
Yamada et al. (28)	65	91.0	New obesity (7), decreased vision (6), hypopituitarism (25)	13.8	94
Chivukula et al. (29)	16	56.2	CSF leak (3), hydrocephalus (2), diabetes insipidus (12), meningitis (2), stroke (3), diplopia (2)	43.8	29
Koutourousiou et al. (30)	17	100.0	CSF leak (2), hypopituitarism (13), diabetes insipidus (11), new obesity (3)	41.1	35
Locatelli et al. (31)	7	100.0	CSF leak (2), new obesity (1), hypopituitarism (3)	14.3	103

*GTR, gross total resection, CSF, cerebrospinal fluid*.

* *Surgical cases in the literature with unavailable data and review articles were not presented in the table*.

### Surgical Exposure and Tumor Resection

The nasal cavity and paranasal sinuses of children have their special anatomic characteristics. The nostrils and nasal passages are relatively narrow, making it difficult to resect the tumor and make pedicled mucosal flaps (9). The procedure of putting in or out of surgical instruments may also increase the damage of the nasal mucosa. In addition, there are differences in sphenoid sinus development with different ages (9, 11, 12). The sphenoid cavity pneumatization first appears on the anterior inferior wall of the sphenoid bone. By the age of 6–7 years, all the anterior walls have been completely pneumatized, and then progresses to the bottom wall of the sphenoid bone, and reaches the sphenoid platform and finally the anterior wall of the sphenoid sella. After 10 years, pneumatization of the sphenoid sinus toward the back of the sphenoid body and clivus can be seen by imaging. Thus, the younger patients are always with poor pneumatization of the sphenoid sinus, which adds more difficulty in exposing the sella through nostrils with neuroendoscopy than adults (32–34). Usually, we chose to gradually expose the floor of the sella in strict accordance with its midline, and after the sellar dura is determined, it is gradually extended to both sides as well as top and bottom. The surgical areas, such as the dorsum sella, clival recess, sellar tuberosity, and sphenoid platform, can be exposed satisfactorily, without extra destruction of the bone at the base of the skull. For those who are unfamiliar with anatomy, the neuronavigation could help to determine the anatomical structures of the sella and its vicinity to avoid unnecessary injury.

After the operation channel is established, the operation space is sufficient, and it does not increase the difficulty of the operation and prolong the operation time compared with the adult operation. However, given the limitation of the narrow nostrils and nasal cavity in children, the patients' selection or surgical indication in children is still unknown and need to be further defined in the future, which may be partly based on the tumor characteristics and patients' age (6, 9). In our surgical group of 8 cases, craniopharyngiomas in children are mainly solid-cystic craniopharyngiomas with obvious calcification, which may be attributed to the enamel type of craniopharyngiomas in this age group. For tumors with obvious cystic changes, decompressing the tumor first by sucking up the liquid oil could achieve more space for resection, with a gentle pull of the tumor capsule after determining the tumor boundary using the neuroendoscopy for close observation. When removing calcified lesions in a piecemeal way, the blood vessels, nerves, and brain tissues around the tumor should avoid being scratched.

According to the QST classification of craniopharyngioma (35–37), the Q-type craniopharyngiomas originate from below the diaphragma sellae, and it is easy to separate the tumor during surgery even if it grows large and invades the hypothalamus, because of the barrier formed by the diaphragm sellae, arachnoid and pial mater between the tumor and the hypothalamus. Some cases with sellar septal tumors should be removed together with the diaphragm to avoid tumor recurrence (35, 38). The S-type craniopharyngiomas on the diaphragm sellae grow from the pituitary stalk to the surrounding area usually with a relatively complete capsule. During the operation, the tumor must be separated from the pituitary stalk along the direction of the pituitary stem. If the pituitary stalk is in the center of the tumor, it must be incised to free the tumor and then try to protect the remaining pituitary stalk (36, 38, 39). For T-type craniopharyngiomas originating from the pars tuberalis, there is only a layer of pial mater between the tumor and the hypothalamus, which may adhere to the nerve tissue in the later stage and is not easy to be separated by surgery, and thus requires to find the boundary between the tumor and the normal tissue (35, 39). For the latter two types, partial tumors of the pituitary stalk cannot be preserved and could be dissected early to reduce the difficulty of tumor resection (35, 37). In addition, excising the tumor along the gliosis zone in the third ventricle may partly ensure that the neural tissue and the third ventricular walls are intact (5). When dealing with the adhesions between the tumor capsule and the optic chiasm, hypothalamus, and third ventricular wall under neuroendoscope, sharp separations should be performed, and a 1–2 mm suction device combined with a right-angle dissector or tumor forceps, with gently stretching or peeling off the tumor under direct views of neuroendoscope, always can remove tumor tissue completely (4, 5). For craniopharyngiomas that extend to the middle and lower clivus, removing the posterior clinoid process and part of the upper clival bone, and opening the membrane of the pituitary gland, and pulling down the tumors through the endoscope to obtain a certain space and angle, especially with the help of angle mirrors, the tumor and surrounding anatomical structures can be observed and then completely removed. Thus, pituitary dysfunction caused by pituitary displacement can be avoided in some cases (1, 4, 6).

Most of the tumors invading the third ventricle can block the CSF circulation and result in obstructive hydrocephalus (1, 3, 5). If patients with hydrocephalus before surgery are in need and have not yet prepared for tumor resection, the Ommaya capsules can be inserted into the ventricle to temporarily relieve high intracranial pressure and to buy time for surgery (3, 7, 14). External ventricular drainage is not recommended because it may increase the postoperative risk of intracranial infection. If the patient's hydrocephalus is not such an emergency, it is wise to directly remove the whole tumor to reconstruct the CSF circulation. During the operation, the Liliquest membrane of the interpeduncular pools can be opened to make the CSF circulation more secure (5). In our surgical group, 4 patients suffered preoperative hydrocephalus that was all caused by a tumor invading the third ventricle, and all of them were improved after the tumor resection. However, for patients with residual tumors that could not be easily removed and thus were left, close follow-up is needed, and generally, gamma knife treatment can be performed 3 months after the operation.

### Skull Base Reconstruction and CSF Leakage

The younger the patients, the poorer postoperative compliance is confronted in children, and the prevention of postoperative CSF leakage is more difficult than that of adults (2, 5, 6, 12). Therefore, reliable skull base reconstruction is a vital part of neuroendoscopic endonasal resection of craniopharyngioma in children to avoid CSF nasal leaks and impossible postoperative intracranial infection (6, 12). During operating, we used the resorbable artificial dura mater to rebuild the first layer of skull base defect, which was inserted into the dura mater to seal the base of the skull, so that the high-flow CSF leak becomes the low-flow CSF leak. If the sphenoid sinus pneumatization is well, the bone flap *in situ* can be made to recover it. If there is no bone flap *in situ*, we attached and paved the pedicled mucosal flap of the nasal septum directly to the skull base defect as the second or third layer of reconstruction, and finally, the iodoform gauze was used to tightly pack and support the mucosal flap for 10–14 days (5, 11, 12). Fortunately, but confidently, there was no CSF effusion in all 8 children. Compared with the same period of adult cases of craniopharyngiomas in our center, the incidence of CSF leak after endoscopic endonasal surgery was relatively lower, which may be attributed to the smaller impact force of CSF flow on the faster-growing mucosal flaps in children to close early the meningeal defect.

### Postoperative Complications and Management of Patients

The children in our surgical cohort had a mild response after resection, without convulsions, coma, and uncontrolled other complications. However, 3 patients presented with transient hyperthermia, and 4 patients had transient hypernatremia and/or hyponatremia after surgery. Particularly, 7 patients had diabetes insipidus after operations, but the degree of polyuria and the remission time was less than those of adults in the same period. The recovery time of children with diabetes insipidus in this group is shorter than that of adults in the same period, which may lie in that most of the tumors were intrasellar and hypothalamic origin in children in this group and thus the pituitary stalks were well protected, while adults in the same period had tumor originating from pituitary stalk. In addition, whether it takes a shorter time to establish paracrine in children than in adults requires further study. Regarding whether patients with craniopharyngiomas should receive neurointensive care, many scholars believe that patients with craniopharyngiomas undergo drastic changes in their physiological and metabolic condition and should be closely monitored (1, 2, 7, 14). However, the children and adults of our center did not receive intensive care after the extended endoscopic endonasal resection. We had considered that all patients were conscious after the operations and could drink and eat on the second day after surgery, and recording the hourly urine volume as well as the physical intake and output volume are necessary and enough, and meanwhile, there may be CSF rhinorrhea or occult CSF rhinorrhea after the expansion of the endonasal approach (2, 3, 14), and the commonly planted bacteria in the intensive care unit may cause a potential risk of intracranial infection or cross-infection due to improper nasal cares. However, the on-duty doctors ought to be guided to deal with the changes in time.

In addition, children with craniopharyngioma need to pay special attention to water and electrolyte balance after surgery (2, 14). High sodium may occur 1–3 days after surgery, so sodium salt should be controlled. On the fourth day or later, low sodium may occur, and the physiological requirement of sodium can be supplemented and corrected in time based on monitoring blood electrolytes. It has been reported that a sharp change in blood sodium (>10 mmol/L) within 24 h is likely to induce epileptiform seizures, especially a sharp drop in blood sodium concentration (40, 41). The mechanism may be due to the rapid occurrence of hyponatremia after reaching a certain threshold, allowing water molecules to quickly enter the cells, and causing brain cell edema and convulsions or coma (14, 41). In this group of cases, 3 cases of transient hypernatremia in the early stage and 2 cases of transient hyponatremia in the late stage were quickly corrected, and no convulsions or coma occurred.

In addition, hypopituitary dysfunctions after endoscopic endonasal surgery were common and should be paid more attention to (2, 3, 14). These endocrine dysfunctions can reduce the quality of life of children and even shorten the life of patients (1, 2). Therefore, the evaluation and treatment of endocrine dysfunction in children before and after surgery, especially subsequently long-term hormone replacement after surgery, is an important principle of the treatment of patients with craniopharyngiomas (2). In our surgical cases in pediatrics, six cases encountered postoperative pituitary–thyroid axis hypofunction, and 7 patients suffered pituitary–adrenal axis dysfunction, and 2 cases had hypogonadism, and 4 cases had decreased growth hormone. At the last time of follow-up, half of the cases gradually improved within 1–3 months after operations, but 4 patients still needed long-term hormone replacement therapy. Furthermore, during the postoperative follow-up, the children's growth, development, and mentality should be regularly assessed, except for the review of tumor recurrence on imaging (2, 3, 14).

## Conclusion

The advantages of the extended endonasal approach for resection of craniopharyngioma by neuroendoscopy are relatively safe and effective for children, with a few light postoperative adverse reactions that do not require intensive care treatment. Due to the poor pneumatization of the sphenoid sinus in pediatrics, surgical drilling of the sellar base along its midline to further expand the exposure of surgical fields was proposed. After the passage is completed, there is no significant difference in removing the tumor when compared with adult endoscopic surgery, and it does not increase the difficulty and time of the operation. After resection, watertight skull base reconstruction should be ensured to reduce postoperative CSF leaks and intracranial infection. Finally, careful postoperative managements of patients are the guarantee for patients who could safely live through the perioperative period. For children who survive for a long time after surgery, hormone level monitoring and hormone replacement therapy are important for postoperative long-term management. Due to the limited surgical cases in this study, the surgical techniques and outcomes of this approach in children need more clinical research in the future.

## Data Availability Statement

The raw data supporting the conclusions of this article will be made available by the authors, without undue reservation.

## Ethics Statement

The studies involving human participants were reviewed and approved by the Ethics Committee of the Affiliated Hospital of Zunyi Medical University. Written informed consent to participate in this study was provided by the participants' legal guardian/next of kin. Written informed consent was obtained from the minor(s)' legal guardian/next of kin for the publication of any potentially identifiable images or data included in this article.

## Author Contributions

DW, LX, and SXie: contributed equally to the article. DW, LX, SXie, and SXiao: wrote the manuscript. DW, LX, SXie, FS, MX, and PW: analyzed and interpreted the patients' data. All authors read and approved the final manuscript.

## Funding

This work was supported by the Guizhou Province Science and Technology Plan (12520000429401122B) and Zunyi Municipal Science and Technology Plan (2019-102).

## Conflict of Interest

The authors declare that the research was conducted in the absence of any commercial or financial relationships that could be construed as a potential conflict of interest.

## Publisher's Note

All claims expressed in this article are solely those of the authors and do not necessarily represent those of their affiliated organizations, or those of the publisher, the editors and the reviewers. Any product that may be evaluated in this article, or claim that may be made by its manufacturer, is not guaranteed or endorsed by the publisher.
